# Effect of climate change on spring wheat yields in North America and Eurasia in 1981-2015 and implications for breeding

**DOI:** 10.1371/journal.pone.0204932

**Published:** 2018-10-17

**Authors:** Alexey Morgounov, Kai Sonder, Aygul Abugalieva, Vijai Bhadauria, Richard D. Cuthbert, Vladimir Shamanin, Yuriy Zelenskiy, Ronald M. DePauw

**Affiliations:** 1 International Maize and Wheat Improvement Center (CIMMYT), Ankara, Turkey; 2 CIMMYT, Texcoco, Mexico; 3 Kazakh Research Institute of Farming, Almaty, Kazakhstan; 4 AAFC Swift Current Research and Development Centre, Saskatchewan, Canada; 5 Omsk State Agrarian University, Omsk, Russia; 6 CIMMYT, Astana, Kazakhstan; 7 Advancing Wheat Technologies, Saskatchewan, Canada; Institute of Genetics and Developmental Biology Chinese Academy of Sciences, CHINA

## Abstract

Wheat yield dynamic in Canada, USA, Russia and Kazakhstan from 1981 till 2015 was related to air temperature and precipitation during wheat season to evaluate the effects of climate change. The study used yield data from the provinces, states and regions and average yield from 19 spring wheat breeding/research sites. Both at production and research sites grain yield in Eurasia was two times lower compared to North America. The yearly variations in grain yield in North America and Eurasia did not correlate suggesting that higher yield in one region was normally associated with lower yield in another region. Minimum and maximum air temperature during the wheat growing season (April-August) had tendency to increase. While precipitation in April-August increased in North American sites from 289 mm in 1981–1990 to 338 mm in 2006–2015 it remained constant and low at Eurasian sites (230 and 238 mm, respectively). High temperature in June and July negatively affected grain yield in most of the sites at both continents. Climatic changes resulted in substantial changes in the dates of planting and harvesting normally leading to extension of growing season. Longer planting-harvesting period was positively associated with the grain yield for most of the locations. The climatic changes since 1981 and spring wheat responses suggest several implications for breeding. Gradual warming extends the wheat growing season and new varieties need to match this to utilize their potential. Higher rainfall during the wheat season, especially in North America, will require varieties with higher yield potential responding to moisture availability. June is a critical month for spring wheat in both regions due to the significant negative correlation of grain yield with maximum temperature and positive correlation with precipitation. Breeding for adaptation to higher temperatures during this period is an important strategy to increase yield.

## Introduction

Wheat and wheat products provide about 20% of protein and 20% of calories consumed per capita [1]. Global wheat area was estimated to be 225 million ha and can be classified into several environments depending on the growth habit, temperature, and moisture availability: spring wheat irrigated/high rainfall, hot and humid; winter wheat irrigated/high rainfall, semi-arid and low rainfall winter and spring wheat [2]. Low rainfall spring wheat is largely produced in high latitude regions of Canada, USA, Kazakhstan, and Russia (above 45° north) with a continental climate. It is a short season crop, growing for approximately 100 days from May to September. Yields are relatively low (1.5–3.0 t/ha) due to the limited growing season, moisture availability, and the impact of abiotic and biotic stresses. Nevertheless, this production environment plays an important role in global food security and wheat prices since most of the grain is traded. The World Wheat Book provides detailed description of spring wheat production and breeding systems in Canada [3], USA [4], European Russia [5], North Kazakhstan and Siberia [6]. Spring wheat production systems of North America and Eurasia are defined by their ecology, climate, technologies, crop varieties, and marketing systems. The hard red spring wheat region in Canada and the USA is situated at slightly lower latitudes than the Eurasian regions of western Siberia and northern Kazakhstan. The North American region is also slightly warmer and receives higher precipitation. Spring wheat varieties in Canada are developed, grown, and traded in eight market classes [7]. These market classes are segregated based on gluten strength, protein content, kernel hardness, and seed color to fit end-user specifications, domestically and internationally. The market classes for spring wheat in Eurasia are very similar to North America and utilize the same criteria. However, production is mainly limited to hard red spring bread wheat. An important difference in the two production systems is a much wider application of zero tillage and conservation agriculture in North America compared to Eurasia. During 1975–2007, the share of wheat in the crop rotation declined while the share of legumes and oil crops increased substantially, and summer fallow acres declined in North America [8]. Zero tillage is gaining popularity in Eurasia where on-farm trials have revealed that it can be a successful dryland cropping method under climate change [9].

Climate change is expected to have large effects on global wheat production: for every 1°C increase in temperature, global wheat yields are predicted to decline by 4.1–6.4% [10]. Wheat grown in warmer regions is likely to experience greater yield losses than that grown in cooler regions, though there is also general agreement that high latitude spring wheat production will benefit from a warmer climate through an extension of the growing period [11]. Simulations of wheat cultivation in the Canadian Prairies estimate that warmer temperatures could lead to yield increases of 26–37% (when elevated CO_2_ concentration was simulated), or up to 15% without considering increased CO_2_ [12]. Wang et al. [13] assessed the impact of climate change on wheat production in 2050 in southern Saskatchewan, Canada. Compared to the baseline (1961–1990), precipitation is projected to increase in every month except July and August while annual mean air temperature is projected to increase by 2.7–3.6°C, depending on the model used. Biomass and grain yield are expected to increase, though heat shocks could cause severe yield reductions. Early seeding must be undertaken to avoid heat damage during the reproductive phase. Using 1961–1990 as a baseline, climate change scenarios projected significantly earlier seeding dates than those currently used [14]. There is no projected reduction in grain yield because precipitation increases during sensitive wheat growth stage. Swinnen et al. [15] estimates that from a biophysical perspective, under different scenarios of land use and climate change, Kazakhstan, Russia and Ukraine could produce an additional 40–110 million tons of wheat compared to current production. Balkovic et al. [16] also predicts that rainfed wheat in Russia has significant potential for growth.

Long-term wheat trials have been successfully utilized to evaluate the effect of weather and climate on adaptation, yield, and related traits. The analysis of spring wheat trials from 1981 till 2002 in Alberta province, Canada, demonstrated that genotype x environment patterns were inconsistent because of complex, highly variable and unpredictable year x location effects [17]. High-yielding and stable varieties were repeatedly selected over years and were popular varieties grown by farmers. The effect of breeding on yield and other important traits of 100 wheat cultivars released in Canada from 1885 to 2012 was studied in seven environments during 2007–2013 [18]. Grain yield was positively correlated with days to maturity and kernel weight but negatively with plant height, lodging, and grain protein content. Days to maturity increased for the Canada Western Red Spring (high protein concentration, strong gluten, high milling yield,) market class. Predominant varieties since about 1997 (AC Barrie, Carberry, and AAC Brandon) have become progressively later maturing than the original standards Neepawa and Katepwa. Increased time to maturity was combined with improved grain yield and shifted the negatively related protein concentration. Variety AC Barrie yields 4.5% more grain combined with 0.4 units more protein, while only being 2.3 days later than Katepwa [19]. Carberry yielded 14% more grain, had 0.3 units more protein concentration, and was 3.5 days later than Katepwa [20]. AAC Brandon yielded 12% more at equal protein concentration while being 3.6 days later than Katepwa [21].

Weather data and agronomic performance of experimental lines and a check variety Thatcher were compiled for six sites in Montana for 1950 to 2007 [22]. Mean annual and March temperature increased significantly at all sites. The planting date has become significantly earlier at a rate of 0.24 d/year. July temperatures increased significantly at two sites and showed negative correlation with grain yield at three sites. Earlier planting due to warmer springs has helped to alleviate negative effects of high temperatures during grain filling period.

This study aimed to evaluate climate change effects on spring wheat development and grain yield at key high latitude breeding sites and its implication on variety enhancement and future production.

## Materials and methods

**Fig 1:**
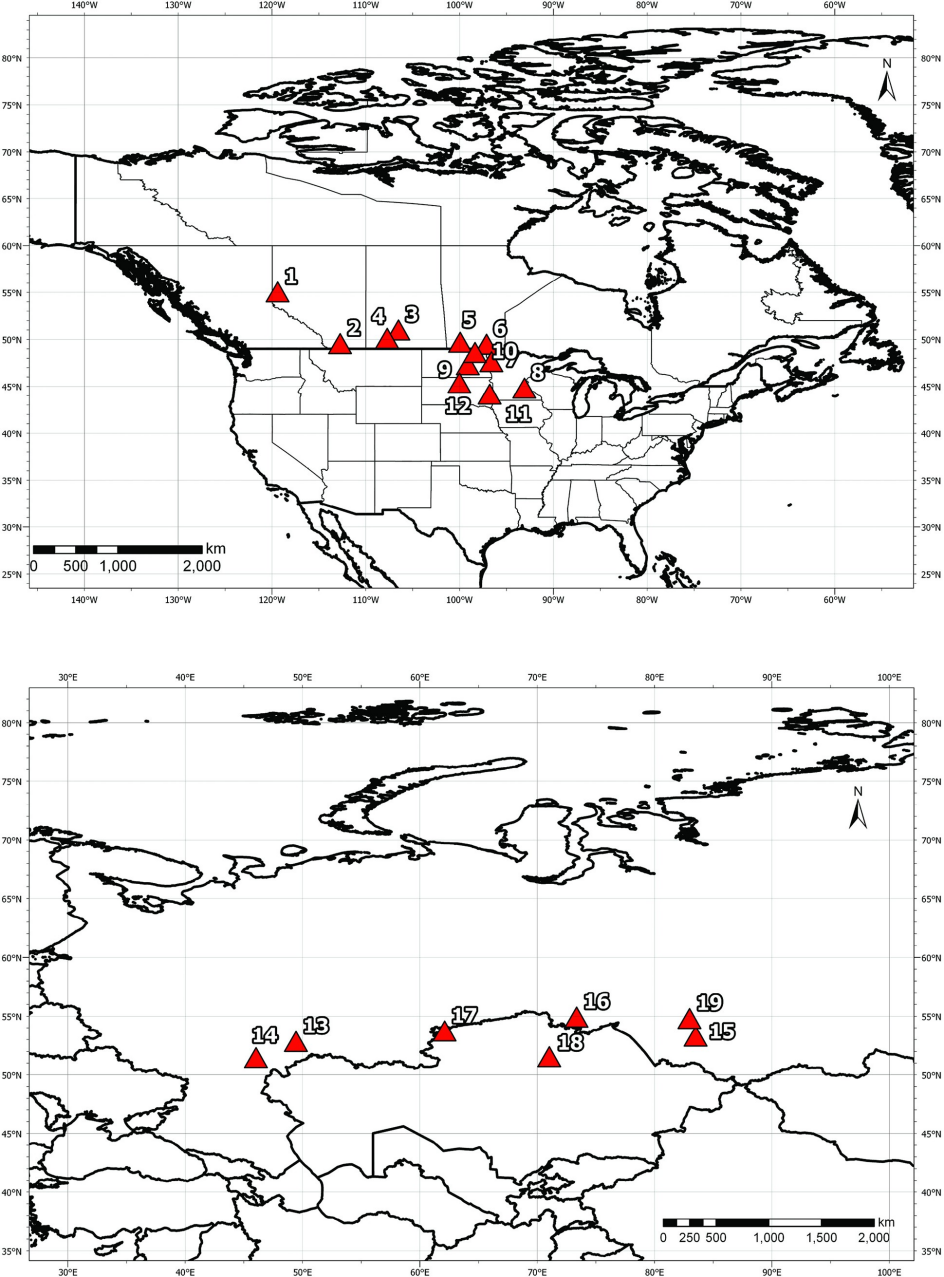
Maps of the study locations.

We selected 19 spring wheat breeding locations across Canada, USA, Kazakhstan, and Russia (Table 1; Fig 1) that represent the diversity of high latitude spring wheat production environments in North America and Eurasia. For all 19 sites, three important weather parameters–monthly average daily minimum temperature (Tmin), monthly average daily maximum temperature (Tmax), and average monthly precipitation–were extracted from the CRU TS3.1 dataset (http://www.cru.uea.ac.uk/) developed by the University of East Anglia [23] from 1981–2015 and used to calculate seasonal and yearly averages. Weather data used in the study are not the actual physical data but approximations based on recordings from nearby weather stations. Evidence of climate change was evaluated for each site by comparing the mean weather parameters values for 1981–1990 versus 2006–2015, and by calculating coefficients of determination (r^2^) of Tmin and Tmax on years during 1981–2015. We assumed that significance of r^2^ indicated significance of weather change over time and thus, evidence for changing climate. The direction of the change was estimated by calculating the regression slope. A similar approach was previously used by Morgounov et al. [24] who analyzed climate change at 35 global winter wheat sites.

**Table 1:** Geographic location of spring wheat breeding sites used in the study, total production spring wheat area and grain yield in the provinces and regions in 2017.

Country/Province or Region	Site#	Site	Institution	Latitude	Longitude	Wheat area, mln ha	Wheat yield, kg/ha
**Canada: Alberta**	1	Beaverlodge, AB	Beaverlodge Research Station, AAFC	55.2072	-119.4284	2.34	3731
	2	Lethbridge, AB	Lethbridge Research Station, AAFC	49.7002	-112.7615		
**Canada: Saskatchewan**	3	Saskatoon, SK	Crop Development Centre, University of Saskatchewan	51.1501	-106.5365	2.80	3120
	4	Swift Current, SK	Swift Current Research and Development Centre, AAFC	50.2608	-107.7395		
**Canada: Manitoba**	5	Brandon, MB	Brandon Research Station, AAFC	49.8500	-99.9505	1.06	4021
	6	Glenlea, MB	Cereal Research Centre, AAFC	49.6352	-97.1361		
**USA: Minnesota**	7	Crookston, MN	University of Minnesota	47.7750	-96.6094	0.47	4505
	8	St. Paul, MN		44.9537	-93.0907		
**USA: North Dakota**	9	Carrington, ND	North Dakota State University	47.4501	-99.1262	2.16	3093
	10	Langdon, ND		48.7603	-98.3681		
**USA: South Dakota**	11	Brookings, SD	South Dakota State University	44.3124	-96.7985	0.39	2757
	12	Selby, SD		45.5066	-100.0320		
**Average North America:**				**48.7135**	**-102.2981**	**9.22**	**3427**
**Russia: Volga**	13	Samara, RU	Samara Agric. Res. Inst.	52.9644	49.4187	0.50	3380
	14	Saratov, RU	Agric. Res. Inst. of South-East	51.5845	46.0063	0.25	2690
Kazakhstan-Russia: West Siberia	15	Barnaul, RU	Altay Agric. Res. Inst.	53.4125	83.5190	2.07	1430
	16	Omsk, RU	Siberian Agric. Res. Inst.	55.0404	73.3604	1.56	1640
	17	Kostanay, KZ	Karabalyk Agric. Exp. Station	53.8540	62.1015	3.67	1240
	18	Astana, KZ	Shortandy Variety Registration Site	51.6645	71.0293	3.72	1180
	19	Novosibirsk, RU	Siberian Crop Production Res. Inst.	54.9182	82.9965	1.07	1870
**Average Eurasia:**				**53.3484**	**66.9188**	**12.84**	**1466**

Spring wheat is normally grown from May until September in Eurasia and from mid-April to late August in North America. Several critical phases of development define a crop’s success or failure: a) crop establishment after planting, which requires moisture and warm temperature (May); b) tillering, stem elongation, and booting (June); c) heading-anthesis (mid-July); and d) grain filling-maturity (July-August). This study focused on weather conditions during these critical phases.

Regional (provinces in Canada, states in USA, and regions in Russia and Kazakhstan) average yield data was obtained using official national statistical sources. Monthly and annual weather variation from 1981 to 2015 at the wheat testing sites was evaluated with annual yield trial data obtained at the same locations. We used data from the following trials (S1 Table):

- Canada: Prairie Recommending Committee for Wheat, Rye and Triticale registration trials (Central Bread Wheat Co-operative test, Parkland Wheat Co-operative test, and the Western Bread Wheat Co-operative test) (uniform germplasm composition across sites in a single year, randomized lattice design with three replications);
- USA: Hard Red Spring Wheat Uniform Regional Performance Nursery (uniform germplasm composition across sites in a single year);
- Russia and Kazakhstan: Breeders advanced yield trials (diverse composition of germplasm across sites in a single year)

Statistical analysis was limited to the calculation of regression parameters, coefficient of correlation (r), coefficient of determination (r^2^), and t-test for comparing the means using MS Excel software.

## Results

This study evaluated spring wheat production area across North America and Eurasia totaling more than 22 million ha (Table 1) and contributing up to 10% of global wheat production. These regions are extremely important for food security because most of the wheat produced is then traded. In 2017, wheat yields in North America were 2–3 times higher than in Eurasia, despite record overall wheat production in Russia. There are several reasons for this difference including environment, overall economic situation, production technologies, and input applications, as well as climate change. Table 2 compares average wheat yields from 1981–1990 and 2006–2015 for both on-station trials and the regional/provincial average. On-station yields in North America demonstrated good progress, increasing by 6.9–69.7% across sites. Farmers production yield in 2006–2015 compared to 1981–1990 demonstrated high yield gain across provinces and states varying from 31 to 65%. Significant yield gain in 35 years was also reflected by significant r^2^ of production yield on years. The yield gap between farmers production and research stations yields narrowed from 1981–90 to 2006–2015 for all the states and provinces of N. America indicating that farmers yield grew faster compared to stations. In Eurasia, by contrast, on-station yields increased by a maximum of 23% at individual sites, and even decreased at two sites. No significant yield gain in production fields was recorded with exception of Saratov. The yield gap in the recent time even widened compared to the 1980s. Overall, both on-farm and on-station historical yield performance in Eurasia was much poorer compared to N. America.

**Table 2:** Changes in on-station and regional production wheat grain yield in 2006–2015 as compared to 1981–1990 and coefficient of determination (r^2^) of grain yield on years.

Site#	Site	On-station average trial^a^ yield (kg ha^-1^)				Province, state, region average yield ^b^ (kg ha^-1^)			
		1981–90	2006–15	% change	r^2^	1981–90	2006–15	% change	r^2^
**1**	**Beaverlodge, AB**	-	3267	-	-	2058	3182	+54.6	0.68***
**2**	**Lethbridge, AB**	2437	3955	+62.3	0.17*^c^				
**3**	**Saskatoon, SK**	3054	3483	+14.0	0.02	1753	2501	+42.7	0.43***
**4**	**Swift Current, SK**	2537	3482	+37.2	0.12*				
**5**	**Brandon, MB**	3627	4306	+18.7	0.11	2113	3005	+42.2	0.50***
**6**	**Glenlea, MB**	3292	4172	+26.7	0.12*				
**7**	**Crookston, MN**	3155	5356	+69.7	0.48***	2743	3591	+30.9	0.33***
**8**	**St. Paul, MN**	2840	4650	+63.7	0.42***				
**9**	**Carrington, ND**	3291	4042	+22.8	0.08	1977	2777	+40.4	0.41***
**10**	**Langdon, ND**	3810	5095	+33.7	0.36***				
**11**	**Brookings, SD**	2532	3678	+45.3	0.31***	1708	2824	+65.3	0.59***
**12**	**Selby, SD**	2750	2942	+6.9	0.00				
**Average N. America**		**3030**	**4036**	**+33.2**	**-**	**2059**	**2980**	**+44.7**	**-**
**13**	**Samara, RU**	2579	1504	-41.3	0.21**	1067	1264	+18.4	0.07
**14**	**Saratov, RU**	2142	1920	-10.4	0.00	1094	1444	+32.0	0.13*
**15**	**Barnaul, RU**	2704	3331	+23.2	0.14*	1269	1158	-8.8	0.01
**16**	**Omsk, RU**	2669	3292	+23.3	0.14*	1350	1426	+5.6	0.01
**17**	**Kostanay, KZ**	2583	3028	+17.2	0.03	1001	1191	+19.0	0.05
**18**	**Astana, KZ**	2355	2534	+7.6	0.02	905	1000	+10.5	0.03
**19**	**Novosibirsk, RU**	3583	2639	-26.3	0.13*	1280	1400	+9.4	0.05
**Average Eurasia**		**2659**	**2607**	**-1.9**	**-**	**1138**	**1269**	**+11.5**	**-**

^a^ The data from the following trials were used in the study: Canada: Prairie Recommending Committee for Wheat, Rye and Triticale registration trials (Central Bread Wheat Co-operative test, Parkland Wheat Co-operative test, and the Western Bread Wheat Co-operative test), uniform composition of germplasm across sites in a single year; USA—Hard Red Spring Wheat Uniform Regional Performance Nursery, uniform composition across sites in a single year; Russia and Kazakhstan–breeders advanced yield trials, diverse composition of germplasm across sites in a single year. Detailed trials description is presented in Supplementary Table 1

^b^ Yield data from official production sources

^c^ *; **; ***—significant at P<0.05; 0.01 and 0.001, respectively.

Year to year variation in farmers’ yields in Canadian provinces and USA states were strongly correlated (coefficients of correlation > 0.6) (S2 Table). High or low yields in one state or province were normally accompanied by high or low yields across North America. The lowest correlation was between the grain yield in Alberta on one hand and in Minnesota and North Dakota on the other hand, respectively 0.47 and 0.56. Yearly yield variation in North American sites did not correspond with most of Eurasian sites. This offers opportunity for stable grain production and mutual compensation when one continent has lower yield while the other produces more. Eurasian sites did not show high and consistent correlation among themselves with the exception of neighboring regions: Samara-Saratov, Kostanay-Astana; Omsk-Novosibirsk, Novosibirsk-Barnaul.

Temporal changes in Tmin and Tmax in 2006–2015 versus 1981–1990 for April-August are presented in Table 3 and r^2^ of Tmin values on years for each month and April to Aug in S3 Table. Tmin tended to increase over the 35 year period analyzed. Significant changes took place in Lethbridge (cooling), St. Paul (warming), and Samara (warming) (Table 3). Tmin increased significantly in St. Paul for the months of June, July, and August, resulting in an increased growing season temperature (S3 Table). Tmin also decreased significantly in Lethbridge during April, May, June, and August, but in Swift Current there was only significant Tmin change in July. For Tmax, significant warming during the whole wheat season was observed only in Samara (Table 3). Tmax increased significantly in April in Barnaul, Astana, and Novosibirsk, while Tmax increased significantly in May at Kostanay and during the year in Astana (S4 Table). Interestingly, the average Tmin for all North American and Eurasian sites for April-August increased by 0.22–0.25°C in 2006–2015 compared to 1981–1990 (Table 3). During the same period, average Tmax at North American sites reduced by 0.32°C, while it increased by 0.54°C across Eurasian sites.

**Table 3:** Changes in mean values for Tmin, Tmax and precipitation for April-August and year in 2006–2015 versus 1981–1990.

Site#	Site	Tmin, °C^1^ (April-August)		Tmax, °C (April-August)		Precipitation, mm (April-August)		Precipitation, mm (Year)	
		1981–90	2006–15	1981–90	2006–15	1981–90	2006–15	1981–90	2006–15
**1**	**Beaverlodge, AB**	6.81	7.12	20.29	20.43	264	270	440	457
**2**	**Lethbridge, AB**	8.56a	7.60b	22.99	22.77	200b	250a	339b	395a
**3**	**Saskatoon, SK**	9.43	9.57	23.69	23.00	205b	246a	326b	378a
**4**	**Swift Current, SK**	8.97	9.65	23.13	22.74	207b	246a	325b	383a
**5**	**Brandon, MB**	10.01	10.44	24.14	23.57	276	314	430b	510a
**6**	**Glenlea, MB**	10.14	10.27	23.90	23.70	286	338	449b	533a
**7**	**Crookston, MN**	11.53	11.25	26.03	25.77	319b	400a	483b	595a
**8**	**St. Paul, MN**	13.72b	15.11a	26.62	26.71	469	518	762	791
**9**	**Carrington, ND**	10.28	10.65	25.25	24.94	295b	348a	435b	518a
**10**	**Langdon, ND**	10.03	9.79	23.81	23.41	290b	356a	427b	516a
**11**	**Brookings, SD**	12.69	13.15	26.54	26.16	366b	440a	557a	644b
**12**	**Selby, SD**	11.67	11.88	26.87	26.26	286	328	402b	464a
***Average N*. *America***		***10*.*32***	***10*.*54***	***24*.*44***	***24*.*12***	***289***	***338***	***448***	***515***
**13**	**Samara, RU**	14.02b	14.44a	25.32b	26.50a	212	188	477	448
**14**	**Saratov, RU**	14.75	15.52	25.90	26.54	194	175	440	438
**15**	**Barnaul, RU**	10.96	10.90	23.28	23.67	230	257	450	470
**16**	**Omsk, RU**	10.38	10.62	22.45	22.52	201	218	372	403
**17**	**Kostanay, KZ**	11.68	12.00	24.66	25.45	205	199	360	366
**18**	**Astana, KZ**	11.36	11.28	24.60	24.97	143	173	272b	327a
**19**	**Novosibirsk, RU**	10.36	10.47	22.47	22.80	426	458	426	458
***Average Eurasia***		***11*.*93***	***12*.*18***	***24*.*10***	***24*.*64***	***230***	***238***	***400***	***416***

^1^—the figures designated with different letters are significantly different at P<0.05.

The biggest temporal change concerned precipitation. Significant increases were observed for yearly precipitation for all North American sites except Beaverlodge and St. Paul (Table 3), as indicated by the r^2^ values (S5 Table). On average, precipitation in 2006–2015 was 15% higher than 1981–1990. The increase was observed in April, May, and June, depending on location. For Eurasian sites, a significant increase was only observed in Astana.

To evaluate dependence of spring wheat grain yield on temperature and precipitation, coefficients of correlations were calculated for the means of on-station grain yield and weather parameters in June, July, and April-August (Table 4). The month of June demonstrated the highest correlation with yield for all three weather parameters. The most critical factor determining grain yield was Tmax in the month of June, with 12 of the 19 sites having significant negative correlation. The April-August period was the second most correlated for all three weather variables, followed by July. Increases in Tmin and Tmax negatively affected yield at two Manitoba sites, Selby (S. Dakota), Samara, Saratov, Kostanay, Astana, and Novosibirsk. Yields in Saskatoon, Swift Current, and Carrington reacted significantly negatively only to increases in Tmax. The yield at two sites in Minnesota, Langdon (N. Dakota), Brookings (S. Dakota), Barnaul and Omsk did not show any significant correlation with temperature or precipitation.

**Table 4:** The coefficients of correlation (r) calculated for monthly Tmin, Tmax, precipitation and on-station grain yield for June, July, April-August at the study breeding sites.

Site#	Site	r calculated for Tmin -grain yield for^a^:			r calculated for Tmax -grain yield for:			r calculated for precip. -grain yield for:		
		June	July	April-August	June	July	April-August	June	July	April-August
**1**	**Beaverlodge, AB**	-0.27	-0.19	0.12	-0.31	-0.10	-0.14	0.38*	-0.05	0.22
**2**	**Lethbridge, AB**	-0.22	0.08	-0.22	-0.37*	-0.11	-0.34	0.41*	-0.01	0.42*
**3**	**Saskatoon, SK**	-0.10	-0.26	-0.31	-0.55**	-0.45*	-0.60***	0.32	-0.02	0.29
**4**	**Swift Current, SK**	-0.16	-0.11	-0.25	-0.46**	-0.57***	-0.64***	0.42*	0.42*	0.65*
**5**	**Brandon, MB**	-0.45*	-0.34	-0.35*	-0.52**	-0.32	-0.50**	0.28	-0.12	0.14
**6**	**Glenlea, MB**	-0.37*	-0.28	-0.41*	-0.37*	-0.15	-0.30	0.12	-0.16	0.09
**7**	**Crookston, MN**	-0.18	-0.10	-0.20	-0.12	0.08	-0.12	0.15	-0.09	0.32
**8**	**St. Paul, MN**	0.20	0.11	0.19	-0.19	0.00	-0.14	0.06	-0.17	-0.01
**9**	**Carrington, ND**	-0.32	-0.29	-0.31	-0.49**	-0.14	-0.39*	0.17	-0.19	0.16
**10**	**Langdon, ND**	-0.21	-0.26	-0.26	-0.11	0.02	-0.06	0.02	-0.16	0.09
**11**	**Brookings, SD**	-0.29	-0.21	-0.29	-0.24	-0.22	-0.29	-0.12	0.04	0.08
**12**	**Selby, SD**	-0.31	-0.49**	-0.36*	-0.49**	-0.51**	-0.52**	0.13	0.24	0.16
**13**	**Samara, RU**	-0.31	-0.46*	-0.50**	-0.40*	-0.50**	-0.56***	0.27	0.12	0.19
**14**	**Saratov, RU**	-0.40*	-0.16	-0.09	-0.47**	-0.20	-0.18	0.36*	-0.02	0.15
**15**	**Barnaul, RU**	0.02	-0.24	0.04	-0.23	-0.21	0.00	0.23	0.00	0.17
**16**	**Omsk, RU**	-0.06	-0.30	-0.03	-0.17	-0.25	-0.23	0.13	-0.16	-0.15
**17**	**Kostanay, KZ**	-0.53**	-0.49*	-0.66***	-0.56***	-0.45**	-0.56***	0.39*	0.13	0.39*
**18**	**Astana, KZ**	-0.36*	-0.41*	-0.33*	-0.47**	-0.41*	-0.43*	0.53**	0.01	0.31
**19**	**Novosibirsk, RU**	-0.38*	-0.14	-0.43*	-0.38*	-0.24	-0.43*	0.11	0.24	0.07

^a^—*; **; ***—significant at P<0.05; 0.01 and 0.001, respectively. Due to yield missing values the r critical values vary across sites.

**Fig 2:**
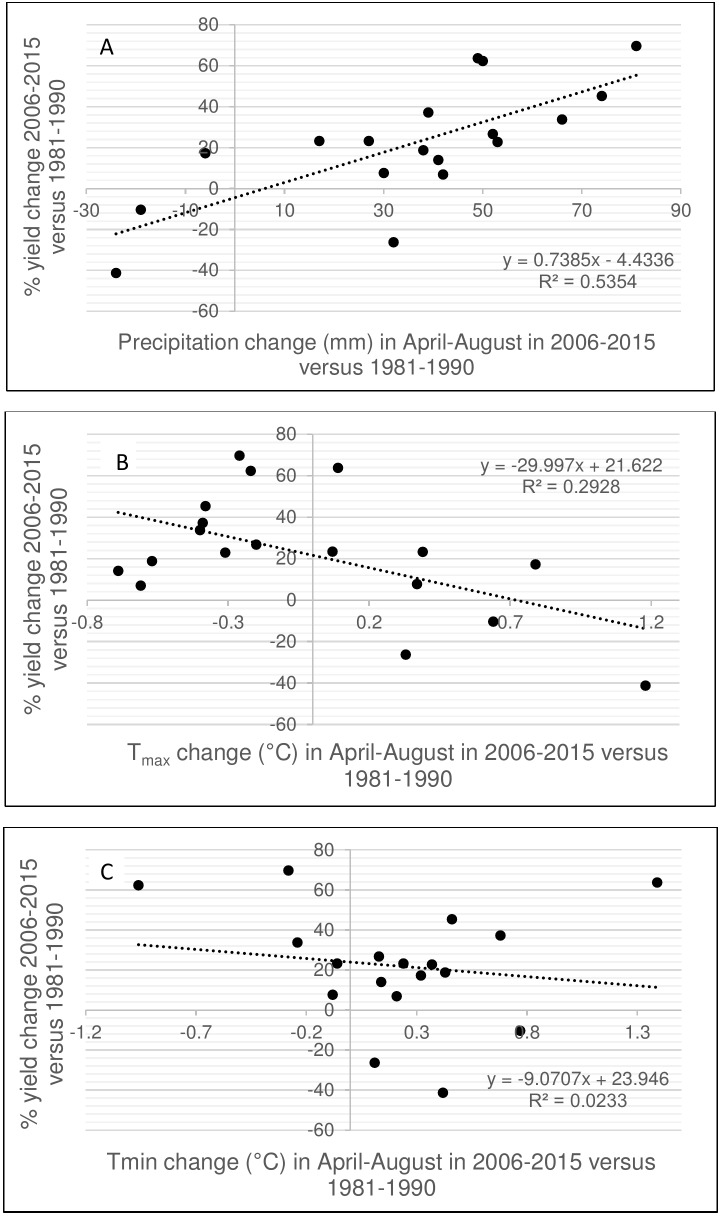
Relationship between the (a) precipitation, (b) Tmax and (c) Tmin change in 2006–2015 versus 1981–1990 and yield change at the breeding sites for the same periods. R^2^ exceeding 0.12 is significant.

Precipitation in June was very critical for spring wheat, with significant positive correlations with grain yield at 6 of 19 sites. Higher dependence of yield on June precipitation was related to the amount of rainfall received during April-August: at drier sites the response was much more pronounced (S1 Fig). Yield differences at individual sites in 2006–2015 versus 1981–1990 were highly depended on precipitation with r^2^ = 0.535 (Fig 2A). Locations with increased rainfall in April-August demonstrated higher yield gains. On the other hand, locations with greater increases in Tmax in 2006–2015 versus 1980–1990 demonstrated reduced yield gains (Fig 2B). Changes in Tmin did not affect grain yield (Fig 2C).

**Fig 3:**
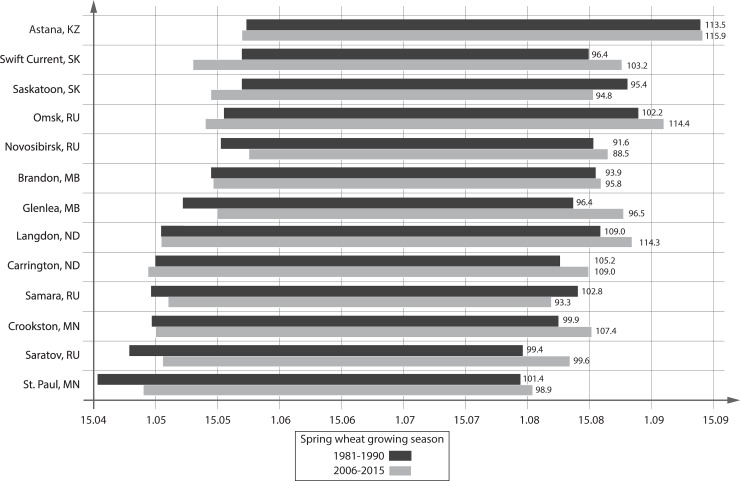
Spring wheat growing season at the breeding sites in 2006–2015 compared to 1981–1990. The horizontal axis reflects dates starting from April 15 with 15 days interval.

Vegetative period duration plays a very important role for a short-season crop like spring wheat. Climate change and yearly weather variations certainly affected the planting and harvesting times throughout the 35 years of this study. All breeding sites in Canada, classify maturity as physiological maturity which occurs when 50% of the spikes have kernels at approximately 30% moisture on a wet weight basis. Actual date of harvest is a function of the passive dry-down weather conditions. Only one location (Samara) demonstrated substantial reduction in planting to harvesting duration from 1981–1990 (102.8 days) to 2006–2015 (93.3 days) (Fig 3, S6 Table). At six sites the vegetative period increased by at least five days in 2006–2015 compared to 1981–1990. At Swift Current, Barnaul, and Omsk planting moved to earlier dates and harvesting to later dates. He et al. [14] anticipated expansion of the growing season at Swift Current. At Langdon, Carrington, and Crookston the planting date did not change but harvesting took place 4–7 days later. At Novosibirsk, Glenlea, Saratov, and St. Paul both planting and harvesting changed by 5–10 days later without change in the duration of growing period. Two locations (Astana and Brandon) demonstrated no change.

**Fig 4:**
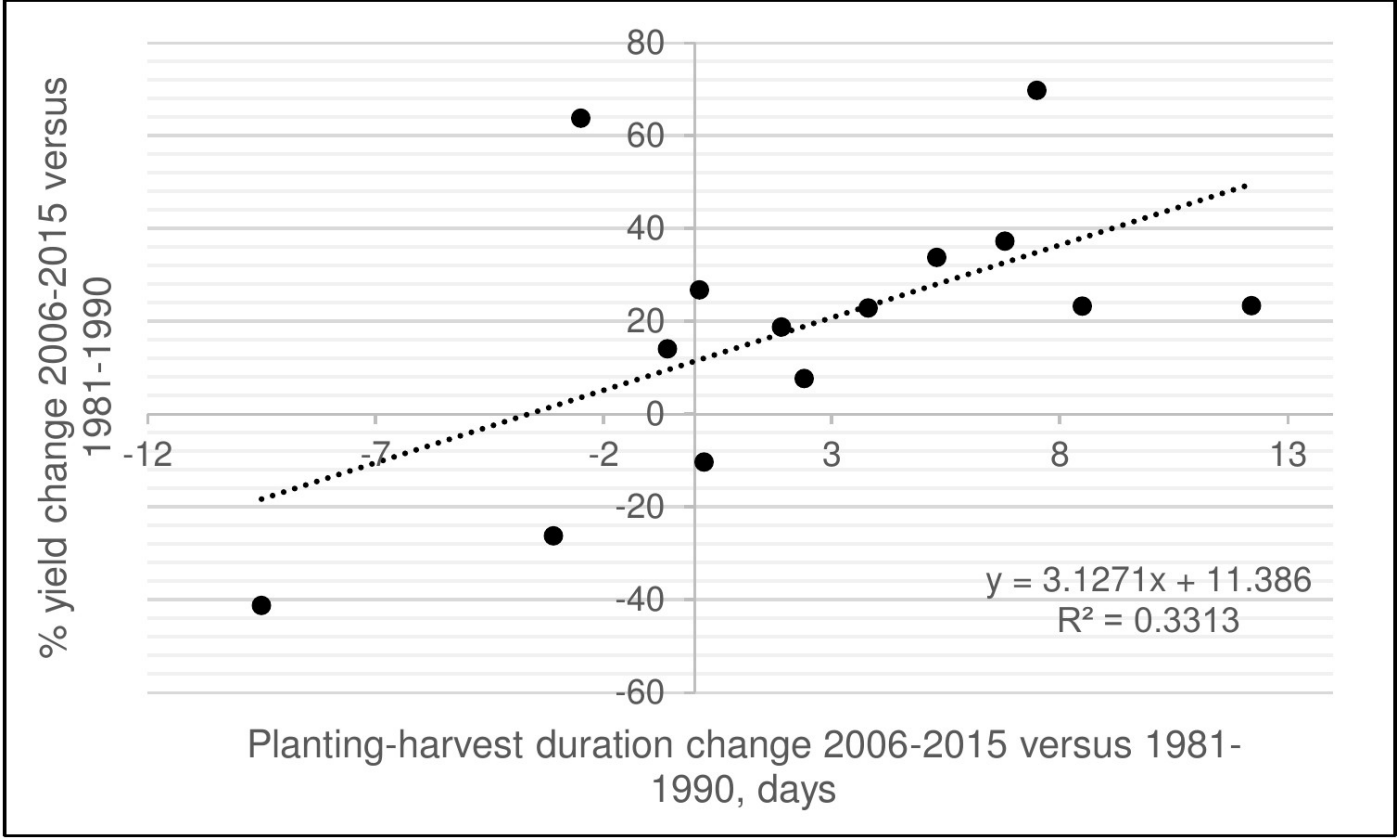
Relationship between the changes (%) in the duration of planting-harvest period in 2006–2015 versus 1981–1990 and % of yield change for the same periods.

For each site, coefficients of correlation were calculated between planting date, harvesting date, and planting-harvesting duration on one hand and grain yield on the other (Table 5). In general, for most sites, later harvesting date as well as increase in planting-harvesting duration was positively and significantly associated with grain yield. Similar results were obtained by Hua et al. [25] for Western Canada. The exceptions were Glenlea, Langdon, Omsk, and Kostanay where no significant correlation with yield was observed. The effect of planting date on grain yield was much less pronounced; only at Brookings did years with later planting result in lower yields due to exposure to high temperatures at maturity. The positive effect of crop cycle duration on grain yield were further supported by plotting changes in planting-harvest period in 2006–2015 versus 1981–1990 against grain yield changes for the same time periods (Fig 4). Sites where planting-harvesting period increased by 3–8 days in 2006–2015 compared to 1981–1990 demonstrated higher yield gains.

**Table 5:** Coefficients of correlation (r) between grain yield and planting date, harvesting date and planting-harvest duration for each breeding site calculated for 1981–2015.

Country/Region	Site#	Site	r with yield^a^:		
			Planting date	Harvest^b^ date	Planting-harvest
**Canada: Alberta**	1	Beaverlodge	-	-	0.47**
	2	Lethbridge	0.30	0.39*	0.25
**Canada: Saskatchewan**	3	Saskatoon	0.15	0.45**	0.40*
	4	Swift Current	-0.08	0.37*	0.55***
**Canada: Manitoba**	5	Brandon	-0.19	0.16	0.51**
	6	Glenlea	0.05	0.24	0.25
**USA: Minnesota**	7	Crookston	-0.07	0.26	0.38*
	8	St. Paul	0.31	0.36*	0.24
**USA: North Dakota**	9	Carrington	0.16	0.72***	0.67***
	10	Langdon	-0.05	0.04	0.08
**USA: South Dakota**	11	Brookings	-0.40*	-	-
	12	Selby	-0.15	-	-
**Russia: Volga**	13	Samara	-0.22	0.28	0.35*
	14	Saratov	0.00	0.43**	0.42*
**Kazakhstan-Russia: West Siberia**	15	Barnaul	-0.27	0.12	0.37*
	16	Omsk	-0.24	0.19	0.26
	17	Kostanay	-0.16	0.13	0.24
	18	Astana	-0.02	0.53***	0.57***
	19	Novosibirsk	0.04	0.35*	0.37*

^a^—*; **; ***—significant at P<0.05; 0.01 and 0.001, respectively. Due to yield missing values the r critical values vary across sites.

^b^ All breeding sites in Canada, report physiological maturity not harvest date. Physiological maturity, maximum dry weight accumulation, has occurred when 50% of the spikes have kernels at approximately 30% moisture on a wet weight basis.

## Discussion

This study analyzed data from 1981 to 2015 to assess the global impacts of climate change. However, comparison of two decades (1981–1990 versus 2006–2015) may be affected by specific factors influencing wheat production. In North America, the 1980s witnessed below long-term precipitation, while 2010–2015 had above average precipitation. Precipitation increases in the 2000s may therefore indicate cyclical change rather than long-term climate change. In Eurasia, wheat production and agriculture underwent the introduction of intensive technologies in the 1980s. The collapse of the Soviet Union and economic crisis that followed greatly affected farming, which experienced a sharp decline and did not recover until the mid-2000s. Other drivers such as wheat prices, biotic and abiotic challenges, government policies, etc. may have also affected wheat production. However, comparing these two decades is still relevant as it considers two different climate scenarios. It is also reinforced by the regression analysis of 35 years of data for key weather and agronomic parameters.

Two regions of spring wheat cultivation evaluated in this study represent very distinct environments, even though both are high latitude with a continental climate. Eurasian spring wheat zone is situated 5° (more than 500 km) further north than the North American region. It is subject to a more continental climate with colder winters, hotter summers, and less precipitation. The Tmin change occurring since 1981 during the wheat cycle (April-August), increasing by 0.25°C in Eurasia and 0.22°C in North America was not significant.

In Eurasia, the wheat cycle Tmax increased by 0.54°C while seasonal and yearly precipitation were essentially unchanged. Planting and harvesting dates both shifted later by 2–3 days, leaving crop cycle duration largely unchanged. However, there are significant regional variations in this respect. At individual research and breeding sites, spring wheat seasons with longer duration and later harvesting dates resulted in higher grain yields. Generally, crop productivity was negatively associated with Tmax in June, July, and April-August, and positively with rainfall in June.

Wheat breeding sites in North America experienced slightly different scenarios. Tmin during April-August increased from 1981–1990 to 2006–2015, while Tmax decreased during the same period. The exposure of spring wheat to heat in North America was therefore slightly reduced. The rainfall during April-August and during agricultural year (October-September) increased. The growing season precipitation, 1981 to 1990, was much below the long term mean while the growing season precipitation in 2010 to 2015 was above the long term mean. This increase in precipitation in would contribute to reduced Tmax. Overall, the climatic changes in North America were more favorable for spring wheat compared to Eurasia due to less exposure to high temperature and more rainfall. As in Eurasia, changes in the planting and harvesting dates were site specific, though there was an overall increase in crop cycle duration by 3–4 days due to later harvesting. For individual sites, the grain yield response to higher temperature, rainfall in spring-summer and growing cycle duration was very similar to Eurasian sites. The analysis of long-term data for Montana spring wheat resulted in similar conclusions [22]. McCaig [26] examined weather variables and durum wheat grain yield at Swift Current for 1946–1995 and reported the highest correlation between weather parameters and grain yield occurred around the end of June through early July, approximately the time of anthesis. The strategy to address the climate change in the Canadian prairies is seeding earlier and choosing cultivars with later maturity dates and longer growing period [14]. Furthermore, because winter temperatures are projected to increase and precipitation in April and May is projected to increase, which are both favorable for winter wheat, another strategy is to increase the planting of winter wheat.

One important difference between North America and Eurasia was air temperature during the growing season. Daily Tmax values represent the highest daytime temperature, which defines the rate of plant growth and development. High Tmax values negatively affects the wheat crop. Daily values of Tmin represent night temperature and are an important indicator of wheat adaptation and grain yield. In Mexico’s Yaqui Valley, historical yields are strongly correlated with Tmin but not Tmax [27]. Trends in recent temperature observations are characterized by greater warming of daily minimum relative to maximum temperatures. The Eurasian breeding sites demonstrated Tmin during April-August at least 1.6°C higher compared to North America, while Tmax was similar in both regions. In this study, the effect of Tmax on grain yield at the breeding sites was much more pronounced than Tmin. However, higher values of Tmin in Eurasia may be one additional factor limiting yield, along with low yearly and seasonal precipitation.

This study was based on data from breeding trials conducted over 35 years, without considering the genetic gains achieved during this period. Several studies have addressed the issue of genetic gains using historical sets of Canadian Western Red Spring Wheat cultivars [28,29], North Dakota Hard Red Spring Wheat [30], and Western Siberia [31]. The breeding progress in these experiments varied from 0.7 to 1.3% per year and was comparable between the programs. However, there is an important difference in the adaptation pattern of spring wheat varieties from North America and Siberia. Trethowan et al. [32] compared the performance of high latitude Canadian, North American, Kazakh, Siberian, Chinese, and Mexican germplasm through a multi-locational trial in these countries. North American germplasm evolved more towards shorter plant stature and day-length insensitivity, compared to Kazakh and Siberian varieties. However, yields of the Kazakh and Siberian germplasm were relatively high, not only in Siberia but also competitive with the Canadian varieties in Canada. The tall stature and day length sensitivity of Siberian germplasm clearly played an important role in adaptating the germplasm to local conditions.

The climatic changes since 1981 and spring wheat responses to variation in air temperature and precipitation suggest several implications for breeding. Gradual warming extends the wheat growing season and new varieties need to match this to utilize their environmental potential. Higher rainfall during the wheat season, especially in North America, will require varieties with higher yield potential responding to moisture availability. June is a critical month for spring wheat in both regions due to the significant negative correlation of grain yield with Tmax and positive correlation with precipitation [26]. June coincides with stem elongation, booting stage, and heading, therefore, heat shock, especially in June and early July can have a detrimental effect on grain yield [26]. Heat shock can also inhibit pollen growth, cause sterility and grain abortion, trigger premature senescence, inhibit kernel development, and cause significant grain yield reductions [33]. Breeding for adaptation to higher temperatures during this period is an important strategy to increase yield. Though the effect of Tmin on grain yield is less pronounced compared to Tmax, the study of its effect on spring wheat yield is well justified. Due to overall low precipitation in Eurasia drought tolerance breeding remains an important objective for the breeding programs.

On a global scale, the North American and Eurasian spring wheat production regions are complementary for stable grain production and food security. Higher yields in one region were normally associated with lower yields in the other. The whole high latitude continental climate spring wheat area certainly represents huge potential for global wheat production. Grise and Kulshreshtha [34] estimated that the area allocated to wheat may continue to decline in Saskatchewan by 2.7–4.6% in various soil zones. Unfortunately, their model did not project additional northern land coming into production as the climate improves for annual crops. In North America, the pace of production gain can be easily sustained, driven by increasing wheat grain demand, especially from wheat importing regions. In Eurasia, yields are so low that they could potentially be doubled or tripled by using modern varieties, technologies, and economic incentives. Spring wheat research and breeding programs in the two regions will benefit tremendously from germplasm exchange and cooperation with emphasis on the climate change challenges and opportunities presented in this paper.

## Supporting information

S1 Table Description of the trials used in the study. (DOCX)

S2 Table Coefficients of correlations between grain yield in different provinces of Canada, states of USA and regions of Russia and Ukraine, 1981–2015. (DOCX)

S3 Table The values of r^2^ for Tmin on years (1981–2015) for each month from April to August, mean Tmin April-August and yearly Tmin. (DOCX)

S4 Table The values of r^2^ for Tmax on years (1981–2015) for each months from April to August, mean Tmax April-August and yearly Tmax. (DOCX)

S5 Table The values of r^2^ for precipitation on years (1981–2015) for each month from April to August, sum of precipitation for April-August and yearly precipitation. (DOCX)

S6 Table Changes in planting date, harvest date and planting-harvest duration at the breeding sites in 2006–2015 compared to 1981–1990. (DOCX)

S1 Fig Relationship between the amount of rainfall in April-August at the study sites and coefficient of correlation between rainfall in June and grain yield. (DOCX)

S1 Data Data underlying the study. (XLSX)

## References

[1] CRP W. Wheat agri-food systems proposal 2017–2022. CGIAR. Research program on Wheat. 2016. 273 p.

[2] Braun H, Atlin G, Payne T. Multi-location testing as a tool to identify plant response to global climate change In: Reynolds M, editor. Climate change and crop production. Wallingford; 2010 p. 115–38.

[3] DePauw R, Hunt T. Canadian Wheat Pool In: Bonjean A, Angus W, editors. The World Wheat Book: A History of Wheat Breeding. Lavoisier; 2001 p. 479–518.

[4] Busch R, Rauch T. US Hard Red Spring Wheat Pool In: Bonjean A, Angus W, editors. The World Wheat Book: A History of Wheat Breeding. Lavoisier; 2001 p. 431–444.

[5] Merezhko A. Wheat pool of European Russia In: Bonjean A, Angus W, editors. The World Wheat Book: A history of wheat breeding. Lavoisier; 2001 p. 257–88.

[6] Morgounov A, Zykin V, Sereda G, Urazaliev R. Siberian and North Kazakhstan Wheat Pool In: Bonjean A, Angus W, editors. The World Wheat Book: A History of Wheat Breeding. Lavoisier; 2001 p.755–772.

[7] McCallum BD, DePauw RM. A review of wheat cultivars grown in the Canadian prairies. Can J Plant Sci. 2008;88(4)(7 1):649–77.

[8] Gan YT, Kutcher R, Menalled F, Lafond L, Brandt S. Crop diversification and intensification with broadleaf crops in cereal-based cropping systems in the Northern Great Plains of North America In: Malhi S, Gan Y, Schoenau J, Lemke R, Liebig M, editors. Recent Trends in Soil Science and Agronomy Research in the Northern Great Plains of North America, Research Signpost, Trivandrum, Kerala, India 2010 p. 277–99.

[9] Kühling I, Redozubov D, Broll G, Trautz D. Impact of tillage, seeding rate and seeding depth on soil moisture and dryland spring wheat yield in Western Siberia. Soil Tillage Res. 2017 7 1;170:43–52.

[10] Liu B, Asseng S, Müller C, Ewert F, Elliott J. Similar estimates of temperature impacts on global wheat yield by three independent methods. Nat Clim Chang. 2016;1130–6.

[11] Sommer R, Glazirina M, Yuldashev T, Otarov A, Ibraeva M, Martynova L, et al Impact of climate change on wheat productivity in Central Asia. Agric Ecosyst Environ. 2013;(178):78–99.

[12] Qian B, De Jong R, Huffman T, Wang H, Yang J. Projecting yield changes of spring wheat under future climate scenarios on the Canadian Prairies. Theor Appl Climatol. 2016 2;123(3):651–69.

[13] Wang H, He Y, Qian B, McConkey B, Cutforth H, McCaig T, et al Climate change and biofuel wheat: A case study of southern Saskatchewan. Canadian journal of plant science. 2012;3(92):421–5.

[14] He Y, Wang H, Qian B, McConkey B, DePauw R. How early can the seeding dates of spring wheat be under current and future climate in Saskatchewan, Canada? PLoS One. 2012;7(10):1–10. 10.1371/journal.pone.0045153 PMC3468606 23094015

[15] Swinnen J, Burkitbayeva S, Schierhorn F, Prishchepov A V., Müller D. Production potential in the “bread baskets” of Eastern Europe and Central Asia. Glob Food Sec. 2017 9 1;14:38–53.

[16] Balkovič J, van der Velde M, Skalský R, Xiong W, Folberth C, Khabarov N, et al Global wheat production potentials and management flexibility under the representative concentration pathways. Glob Planet Change. 2014 11 1;122:107–21.

[17] Navabi A, Yang R, Helm J, Spaner D. Can spring wheat-growing megaenvironments in the northern Great Plains be dissected for representative locations or niche-adapted genotypes? Crop Sci. 2006;46(3):1107–16.

[18] Iqbal M, Moakhar N, Strenzke K, Haile T, Pozniak C, Hucl P, et al Genetic improvement in grain yield and other traits of wheat grown in western Canada. Crop Sci. 2016;56(2):613–24.

[19] McCaig T, DePauw R, Clarke J, Mcleod J, Fernandez M, Knox R. AC Barie hard red spring wheat. Can J Plant Sci. 1996;76(2):337–9.

[20] DePauw RM, Knox RE, McCaig TN, Clarke FR, Clarke JM. Carberry hard red spring wheat. Can J Plant Sci. 2011;91(3):529–34.

[21] Cuthbert R, DePauw R, Knox R, Singh A, McCaig T, McCallum B, et al AAC Brandon Hard Red Spring Wheat. Can J Plant Sci. 2017;97(2):393–401.

[22] Lanning SP, Kephart K, Carlson GR, Eckhoff JE, Stougaard RN, Wichman DM, et al Climatic Change and Agronomic Performance of Hard Red Spring Wheat from 1950 to 2007. Crop Sci. 2010;50(3):835–41.

[23] Mitchell TD, Jones PD. An improved method of constructing a database of monthly climate observations and associated high-resolution grids. Int J Climatol. 2005;25(6):693–712.

[24] Morgounov A, Haun S, Lang L, Martynov S, Sonder K. Climate change at winter wheat breeding sites in central Asia, eastern Europe, and USA, and implications for breeding. Euphytica. 2013;194(2):277–92.

[25] Chen H, Moakhar NP, Iqbal M, Pozniak C, Hucl P, Spaner D. Genetic variation for flowering time and height reducing genes and important traits in western Canadian spring wheat. Euphytica. 2016;208(2):377–90.

[26] McCaig TN. Temperature and precipitation effects on durum wheat grown in southern Saskatchewan for fifty years. Can J Plant Sci. 1997;77(2):215–23.

[27] Lobell DB, Ortiz-Monasterio JI. Impacts of day versus night temperatures on spring wheat yields. Agron J. 2007;99(2):469–77.

[28] DePauw RM, Knox RE, Clarke FR, Wang H, Fernandez MR, Clarke JM, et al Shifting undesirable correlations. Euphytica. 2007;157(3):409–15.

[29] Thomas JB, Graf RJ. Rates of yield gain of hard red spring wheat in western Canada. Can J Plant Sci. 2014;94(1):1–13.

[30] Underdahl JL, Mergoum M, Ransom JK, Schatz BG. Agronomic traits improvement and associations in hard red spring wheat cultivars released in North Dakota from 1968 to 2006. Crop Sci. 2008;48(1):158–66.

[31] Morgounov A, Zykin V, Belan I, Roseeva L, Zelenskiy Y, Gomez-Becerra HF, et al Genetic gains for grain yield in high latitude spring wheat grown in Western Siberia in 1900–2008. F Crop Res. 2010;117(1):101–12.

[32] Trethowan RM, Morgunov A, He Z, DePauw R, Crossa J, Warburton M, et al The global adaptation of bread wheat at high latitudes. Euphytica [Internet]. 2006 12;152(3):303–16. Available from: 10.1007/s10681-006-9217-1

[33] Wang H, Lemke R, Goddard T, Sprout C. Tillage and root heat stress in wheat in central Alberta. Can J Soil Sci. 2007;87(1):3–10.

[34] Grise J, Kulshreshtha S. Farmers’ choice of crops in Canadian prairies under climate change: An econometric analysis. J Earth Sci Clim Change. 2015;07(02):332.

