# Supplementary material for: Effect of climate change on spring wheat yields in North America and Eurasia in 1981-2015 and implications for breeding
Source: PLoS One. 2018 Oct 17;13(10):e0204932. doi: 10.1371/journal.pone.0204932 (PMC6192627; doi:10.1371/journal.pone.0204932)
Supplement: S1 Table — (DOCX) [file pone.0204932.s001.docx]

**S1 Table. Description of the trials used in the study.**

| Site# | Site name, province/state/region, country | Trial name | Number of entries | Replication | Years with missing data |
| --- | --- | --- | --- | --- | --- |
| 1 | Beaverlodge, Alberta, Canada | PRCWRT^a^ Registration Trials | 25- 30 | 3 | 1983; 1986; 1988; 1990; 2013 |
| 2 | Lethbridge, Alberta, Canada |  |  |  | 1985; 1988; 2005; 2013 |
| 3 | Saskatoon, Saskatchewan, Canada |  |  |  | 1988; 2002 |
| 4 | Swift Current, Saskatchewan, Canada |  |  |  | - |
| 5 | Brandon, Manitoba, Canada |  |  |  | 1992; 2000; 2011 |
| 6 | Glenlea, Manitoba, Canada |  |  |  | 1994; 2004; 2007; 2013; 2014; 2015 |
| 7 | Crookston, Minnesota, USA | HRSWURN^b^ Performance Nursery | 32-40 | 3 | 2011; 2012 |
| 8 | St. Paul, Minnesota, USA |  |  |  | 2011; 2012 |
| 9 | Carrington, North Dakota, USA |  |  |  | 1991; 2011; 2012 |
| 10 | Langdon, North Dakota, USA |  |  |  | 2011; 2012 |
| 11 | Brookings, South Dakota, USA |  |  |  | 1982; 2011; 2012 |
| 12 | Selby, South Dakota, USA |  |  |  | 1982; 1986; 1997; 2011; 2012 |
| 13 | Samara, Russia | Breeding programs advanced yield trials and Registration Trial in Astana | 15-30 | 4 | - |
| 14 | Saratov, Russia |  |  |  | - |
| 15 | Barnaul, Russia |  |  |  | - |
| 16 | Omsk, Russia |  |  |  | - |
| 17 | Kostanay, Kazakhstan |  |  |  | 2006 |
| 18 | Astana, Kazakhstan |  |  |  | - |
| 19 | Novosibirsk, Russia |  |  |  | 1997-2002; 2005 |

^a^ Prairie Recommending Committee for Wheat, Rye and Triticale

^b^ Hard Red Spring Wheat Uniform Regional Performance Nursery
