# Supplementary material for: Effect of climate change on spring wheat yields in North America and Eurasia in 1981-2015 and implications for breeding
Source: PLoS One. 2018 Oct 17;13(10):e0204932. doi: 10.1371/journal.pone.0204932 (PMC6192627; doi:10.1371/journal.pone.0204932)
Supplement: S2 Table — (DOCX) [file pone.0204932.s002.docx]

**S2 Table.**  **Coefficients of correlations between grain yield in different provinces of Canada, states of USA and regions of Russia and Ukraine, 1981-2015.**

| Regions | Saskatchewan | Manitoba | Minnesota | N. Dakota | S. Dakota | Samara | Saratov | Kostanay | Omsk | Astana | Barnaul | Novosibirsk |
| --- | --- | --- | --- | --- | --- | --- | --- | --- | --- | --- | --- | --- |
| Alberta | 0.86*** ^a^ | 0.64*** | 0.47** | 0.56*** | 0.70*** | 0.19 | 0.30 | 0.10 | -0.03 | 0.11 | -0.29 | -0.07 |
| Saskatchewan |  | 0.76*** | 0.55*** | 0.69*** | 0.70*** | 0.24 | 0.22 | 0.17 | -0.03 | 0.20 | -0.23 | -0.08 |
| Manitoba |  |  | 0.84*** | 0.89*** | 0.82*** | 0.36* | 0.34* | 0.21 | 0.06 | 0.19 | -0.07 | 0.15 |
| Minnesota |  |  |  | 0.87*** | 0.82*** | 0.25 | 0.19 | 0.21 | 0.11 | 0.22 | 0.10 | 0.23 |
| N. Dakota |  |  |  |  | 0.89*** | 0.28 | 0.24 | 0.11 | 0.13 | 0.16 | 0.02 | 0.20 |
| S. Dakota |  |  |  |  |  | 0.28 | 0.34* | 0.11 | 0.14 | 0.12 | -0.08 | 0.22 |
| Samara |  |  |  |  |  |  | 0.70*** | 0.38* | 0.31 | 0.32 | 0.11 | 0.28 |
| Saratov |  |  |  |  |  |  |  | 0.35* | 0.17 | 0.23 | 0.03 | 0.03 |
| Kostanay |  |  |  |  |  |  |  |  | 0.32 | 0.71*** | 0.03 | 0.19 |
| Omsk |  |  |  |  |  |  |  |  |  | 0.39* | 0.38* | 0.56*** |
| Astana |  |  |  |  |  |  |  |  |  |  | 0.20 | 0.29 |
| Barnaul |  |  |  |  |  |  |  |  |  |  |  | 0.64*** |

^a^ - *; **; *** - significant at P<0.05; 0.01 and 0.001, respectively
