# Supplementary material for: Effect of climate change on spring wheat yields in North America and Eurasia in 1981-2015 and implications for breeding
Source: PLoS One. 2018 Oct 17;13(10):e0204932. doi: 10.1371/journal.pone.0204932 (PMC6192627; doi:10.1371/journal.pone.0204932)
Supplement: S5 Table — (DOCX) [file pone.0204932.s005.docx]

**S5 Table.** **The values of r^2^ for precipitation on years (1981-2015) for each month from April to August, sum of precipitation for April-August and yearly precipitation.**

| Site# | Site | r^2^ calculated for precipitation on years for 1981-2015 | | | | | | |
| --- | --- | --- | --- | --- | --- | --- | --- | --- |
|  |  | April | May | June | July | August | Apr.-Aug. | Year |
| 1 | Beaverlodge, AB | 0.02 | 0.06 | 0.04 | 0.07 | 0.09 | 0.00 | 0.03 |
| 2 | Lethbridge, AB | 0.29*^**a^ | 0.00 | 0.12* | 0.00 | 0.00 | 0.11 | 0.14* |
| 3 | Saskatoon, SK | 0.20* | 0.01 | 0.15* | 0.01 | 0.01 | 0.09 | 0.11 |
| 4 | Swift Current, SK | 0.32*** | 0.00 | 0.13* | 0.05 | 0.01 | 0.07 | 0.12* |
| 5 | Brandon, MB | 0.14* | 0.08 | 0.03 | 0.00 | 0.00 | 0.05 | 0.16* |
| 6 | Glenlea, MB | 0.22** | 0.26*** | 0.00 | 0.00 | 0.00 | 0.06 | 0.10 |
| 7 | Crookston, MN | 0.12* | 0.14* | 0.04 | 0.00 | 0.00 | 0.14* | 0.19** |
| 8 | St. Paul, MN | 0.02 | 0.03 | 0.03 | 0.01 | 0.01 | 0.05 | 0.00 |
| 9 | Carrington, ND | 0.05 | 0.09 | 0.01 | 0.00 | 0.04 | 0.11 | 0.15* |
| 10 | Langdon, ND | 0.13* | 0.13* | 0.00 | 0.01 | 0.04 | 0.12* | 0.13* |
| 11 | Brookings, SD | 0.05 | 0.08 | 0.05 | 0.02 | 0.04 | 0.11 | 0.10 |
| 12 | Selby, SD | 0.01 | 0.05 | 0.03 | 0.03 | 0.02 | 0.05 | 0.08 |
| 13 | Samara, RU | 0.01 | 0.08 | 0.01 | 0.02 | 0.04 | 0.01 | 0.01 |
| 14 | Saratov, RU | 0.00 | 0.00 | 0.00 | 0.03 | 0.00 | 0.00 | 0.01 |
| 15 | Barnaul, RU | 0.02 | 0.00 | 0.02 | 0.00 | 0.06 | 0.02 | 0.01 |
| 16 | Omsk, RU | 0.05 | 0.00 | 0.01 | 0.00 | 0.01 | 0.01 | 0.04 |
| 17 | Kostanay, KZ | 0.02 | 0.00 | 0.01 | 0.00 | 0.00 | 0.00 | 0.02 |
| 18 | Astana, KZ | 0.03 | 0.00 | 0.01 | 0.11 | 0.06 | 0.10 | 0.22** |
| 19 | Novosibirsk, RU | 0.02 | 0.01 | 0.03 | 0.07 | 0.06 | 0.04 | 0.07 |

^a^ - *; **; *** - significant at P<0.05; 0.01 and 0.001, respectively.
