# Supplementary material for: Effect of climate change on spring wheat yields in North America and Eurasia in 1981-2015 and implications for breeding
Source: PLoS One. 2018 Oct 17;13(10):e0204932. doi: 10.1371/journal.pone.0204932 (PMC6192627; doi:10.1371/journal.pone.0204932)
Supplement: S6 Table — (DOCX) [file pone.0204932.s006.docx]

**S6 Table.** **Changes in planting date, harvest date and planting-harvest duration at the breeding sites in 2006-2015 compared to 1981-1990.**

| Site | Planting date | | Harvest date^a^ | | Plant-Harvest, days | |
| --- | --- | --- | --- | --- | --- | --- |
|  | 1981-90 | 2006-15 | 1981-90 | 2006-15 | 1981-90 | 2006-15 |
| Saskatoon, SK | May 20 | May 13 | August 23 | August 16 | 95.4 | 94.8 |
| Swift Current, SK | May 20 | May 10 | August 15 | August 21 | 96.4 | 103.2 |
| Brandon, MB | May 13 | May 14 | August 17 | August 18 | 93.9 | 95.8 |
| Glenlea, MB | May 6 | May 15 | August 11 | August 21 | 96.4 | 96.5 |
| Crookston, MN | April 29 | May 1 | August 8 | August 16 | 99.9 | 107.4  |
| St. Paul, MN | April 16 | April 25 | July 28 | August 2 | 101.4 | 98.9 |
| Carrington, ND | May 1 | April 28 | August 7 | August 15 | 105.2 | 109.0 |
| Langdon, ND | May 3 | May 3 | August 18 | August 25 | 109.0 | 114.3 |
| **Average N. America** | **May 6** | **May 6** | **August 12** | **August 17** | **99.7** | **102.5** |
| Samara, RU | April 30 | May 4 | August 11 | August 5 | 102.8 | 93.3 |
| Saratov, RU | April 23 | May 3 | July 31 | August 10 | 99.4 | 99.6 |
| Barnaul, RU | May 14 | May 9 | August 25 | August 29 | 102.9 | 111.4 |
| Omsk, RU | May 17 | May 13 | August 27 | Sept. 5 | 102.2 | 114.4 |
| Novosibirsk, RU | May 16 | May 21 | August 16 | August 18 | 91.6 | 88.5 |
| Astana, KZ | May 22 | May 20 | Sept. 12 | Sept. 13 | 113.5 | 115.9 |
| **Average Eurasia** | **May 10** | **May 12** | **August 20** | **August 23** | **102.1** | **103.8** |

^a^ - Maturity in Canada is classified as physiological maturity (30% to 35% on a wet weight basis).
