# Supplementary material for: Effect of climate change on spring wheat yields in North America and Eurasia in 1981-2015 and implications for breeding
Source: PLoS One. 2018 Oct 17;13(10):e0204932. doi: 10.1371/journal.pone.0204932 (PMC6192627; doi:10.1371/journal.pone.0204932)
Supplement: S1 Fig — (DOCX) [file pone.0204932.s007.docx]

**S1 Fig. Relationship between the amount of rainfall in April-August at the study sites and coefficient of correlation between rainfall in June and grain yield.**
